# Characterizing Computer Access Using a One-Channel EEG Wireless Sensor

**DOI:** 10.3390/s17071525

**Published:** 2017-06-29

**Authors:** Alberto J. Molina-Cantero, Jaime Guerrero-Cubero, Isabel M. Gómez-González, Manuel Merino-Monge, Juan I. Silva-Silva

**Affiliations:** 1 Departamento de Tecnología Electrónica, ETS Ingeniería Informática, Universidad de Sevilla, Campus de Reina Mercedes, Sevilla 41012, Spain; jaimereben@gmail.com (J.G.-C.); igomez@us.es (I.M.G.-G.); manmermon@dte.us.es (M.M.-M.); 2 ASPACE Sevilla, Dos Hermanas, Sevilla 41704, Spain; nacho.aspace.2@gmail.com

**Keywords:** cerebral palsy, attention, brain computer interface, wireless EEG sensor, linear discriminant analysis

## Abstract

This work studies the feasibility of using mental attention to access a computer. Brain activity was measured with an electrode placed at the Fp1 position and the reference on the left ear; seven normally developed people and three subjects with cerebral palsy (CP) took part in the experimentation. They were asked to keep their attention high and low for as long as possible during several trials. We recorded attention levels and power bands conveyed by the sensor, but only the first was used for feedback purposes. All of the information was statistically analyzed to find the most significant parameters and a classifier based on linear discriminant analysis (LDA) was also set up. In addition, 60% of the participants were potential users of this technology with an accuracy of over 70%. Including power bands in the classifier did not improve the accuracy in discriminating between the two attentional states. For most people, the best results were obtained by using only the attention indicator in classification. Tiredness was higher in the group with disabilities (2.7 in a scale of 3) than in the other (1.5 in the same scale); and modulating the attention to access a communication board requires that it does not contain many pictograms (between 4 and 7) on screen and has a scanning period of a relatively high $t_{scan}\approx$ 10 s. The information transfer rate (ITR) is similar to the one obtained by other brain computer interfaces (BCI), like those based on sensorimotor rhythms (SMR) or slow cortical potentials (SCP), and makes it suitable as an eye-gaze independent BCI.

## 1. Introduction

Communication is vital for human beings. A system allowing people with disabilities to access a computer or a communication system reliably, with little effort and as fast as possible, would be highly beneficial. There are several devices on the market and scientific papers which translate user intentionality into events. The simplest and one of the most extended is based on a binary switch (on/off contacts). A good survey for assistive devices can be found in [1].

Most organizations that give care to people with disabilities use such devices on a massive scale so that they can use software applications, particularly those based on scanning methods, by simply connecting the switch to an adapted device which translates user movements into software selections (mouse clicks, enter keystroke, etc.). For people with severe disabilities, such as those with hypothonic, ataxic neuro-muscular diseases or ALS (amyotrophic lateral sclerosis), these simple devices are still very difficult to use. For them, BCI (brain computer interfaces) systems could be a feasible alternative.

BCI systems [2,3] are based on recording cortical neuronal activity, and one way to achieve this is by means of EEG (Electro-Encephalo-Graphy), which requires several electrodes placed on the scalp. One possible drawback with these systems is their cost, which prevents most people with disabilities from acquiring it. Nevertheless, some companies, such as Emotiv (San Francisco, CA, USA) and Neurosky (San Jose, CA, USA) have released their wireless BCI headsets (Emotiv Epoc, Neurosky mindwave, ⋯) for entertainment uses such as brain gaming and mind monitoring with affordable prices for the consumers. Emotiv has up to 14 channels covering all of the cerebral lobes and the two hemispheres, and it has also been studied as a potential BCI system for people with disabilities [4]. NeuroSky mindwave is cheaper than the Emotiv epoc, and it only has one channel placed at the pre-frontal left position, Fp1. In [5], a comparison was carried out between both low-cost systems to detect cognitive loads. The authors found that Emotiv provided better results but recognized the advantages of Neurosky because it is more user-friendly, easier to setup and maintain. Neurosky’s devices have been used in scientific research, for example, as low-cost EEG-based sleep or drowsiness detection systems [6,7], to measure the subject’s workload in [8,9] during the performance of different tasks, as an emergency call system [10], to assist people with reduced mobility in the school inclusion process [11], to categorize elite’s archers capability of attention control during shooting process [12] or for detecting or recognizing emotional [13,14,15], attentional [16,17,18] or relaxation [19,20] states. Neurosky mindwave delivers information that we can classify in three levels of processing. From lowest to higher levels, they are: raw EEG signal, power bands and eSense, which includes propietary meters for attention and meditation. Power bands and eSense signals help reduce the processing of the raw signals in external devices and allow for using digital systems with low computation resources.

This work looks into the feasibility of using cognitive skills, like attention, to control a system in a binary way (on, -high attention-, off -low attention-), such as a switch. The experiment was performed first by normally developed people and then by people with cerebral palsy (CP) with severe motor dysfunction but with good intellectual capabilities. It includes the eSense attention signal and the power bands as well. Prior work can be found in [21], wherein people with CP took part in a experiment in which they had to control their attention and relaxation signals to play with different games. In those games, the players had to reach a certain level of attention and/or to keep it over a preset value to make the game advance. Results showed that the participants with CP could control their attentional level in a similar way to people without disabilities. However, using the attention to access a computer (i.e., to a communication application) requires a little more complex ability: to keep the attention low/high for a while and being volitionally able to switch between them. In this work, we first investigated the accuracy in detection of the high/low levels of attention by means of a linear discriminant classifier and then proposed a method to estimate the accuracy of using a communication system.

Section 2 briefly explains the fundamentals of attention and some techniques used to measure it. Section 3 describes the methodology followed in experimentation, Section 4 and Section 5 the results and how we used them to characterize the computer access, and, finally, Section 6 and Section 7 contain the discussion and the conclusions respectively.

## 2. Measuring the Attention

**Figure 1:**
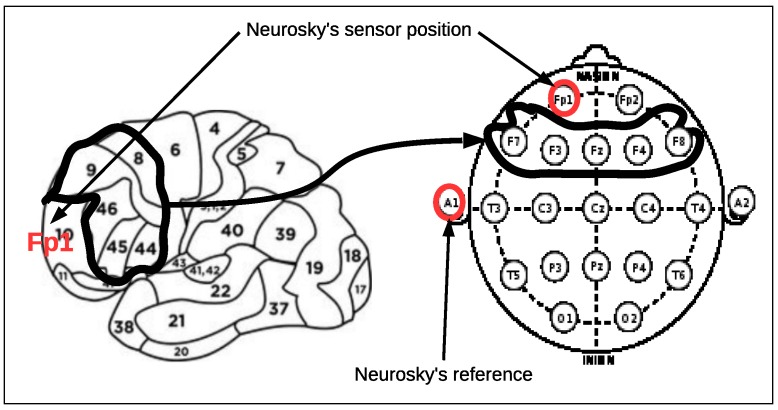
On the left, the lateral prefrontral cortex (LPFC) and the Brodmann areas related to it. On the right, the electrodes’ placement in the 20-20 international system mainly affected by LPFC. The position of the Neurosky’s electrode is also shown.

Attention is the ability to focus continuously on a particular action, thought or object. Attention is controlled by both cognitive top-down factors, such as knowledge, expectation and current goals, and bottom-up factors that reflect sensory stimulation. For example, brightly colored or fast moving objects are often important and are therefore salient stimuli (bottom-up). However, intelligent behavior depends on top-down control signals that can modulate sensory processing in favor of inputs more relevant to achieving long-term goals. Neurophysiological studies have begun to distinguish the circuitry, within a shared frontal-parietal network, that guides top-down and bottom-up attention. Namely, cognitive factors in attention (top-down) arises from the lateral prefrontral cortex (LPFC) [22,23] (see Figure 1).

Several physiological markers can be used to indicate attention levels: eye tracking is a popular and a simple approach for estimating the focus of visual attention; eye pupil dilation, which is proportional to attention; the blinking rate, which decreases as attention level increases [24,25] and the modulation of the EEG activity.

From a temporal point of view, attention makes EEG signals more complex, so its measurement could be based on its fractal dimension. Several works have shown the reliability of such an approach [26,27,28,29]. There have also been some works on the effects that attention or cognitive skills have on power bands. In general, the α band increases as the difficulty of the task diminishes or after task practice, suggesting that fewer cortical resources are required [30]. In the same work, increases in θ suggested that focusing attention or increasing the memory load require more effort. A prolonged period of cognitive activity leads to mental fatigue which is associated with an increment in frontal θ and α activity [31], but after α power reaches a value, θ goes on increasing. In [32], an increment was reported in δ activity related to attention to internal processing during the performance of a mental task. The use of the ratio between frequency bands like θ/β, known as TBR, has also been reported as an indicator for attention deficit disorder (ADD) or hyperactivity disorder (ADHD) patients [33]. TBR is increased in frontocentral children suffering from attention deficit disorders.

Some papers have shown the feasibility of detecting different relaxation and attention states using a reduced number of electrodes. For instance, in [34], two sets of electrodes were used to control the position of an object on a computer screen by means of concentration. One set had 16 electrodes covering different areas and hemispheres on the scalp. The other set had only one electrode placed at the Pz position. Results showed that a high percentage of participants (70%) in the experiment could control the game using only one electrode. Such a percentage increased when the first set of electrodes was used. In [35], two electrodes at positions Fp1 and Fp2 were used to detect the relaxation level. Authors reported that the sum of α+θ, and α+β+θ were good indexes for the measurement of relaxation. In [36], five different bipolar configurations of two electrodes were investigated during attention exercises. Results showed that EEG rhythms were observed with more amplitude in two EEG channels: Fp1-A1 and FP1-T3. They adopted the configuration Fp1-A1 because those positions are free of hair, which allows an easy electrode placement (these are the positions used in the Neurosky mindwave). They also found that the α, β and γ rhythms presented significant differences (*p* < 0.05) between low- and high-attention levels. For this reason, they proposed an index, named attention power (AP), based on the sum of the power α and β bands to control a game. Eighty percent of the subjects found correlation between his/her attention level and the effect exerted over the game.

Neurosky’s manufacturer states that attention signal has more emphasis on beta wave, but the exact algorithm has not been published.

Nevertheless, several studies have shown the feasibility of using this device for measuring the attention level. In [16], it was shown that there is a positive correlation between the reported attention level of this device and the self-reported attention levels of the participants in an experiment that analyzed the Neurosky usability in an assessment exercise. In [37], the single-channel EEG device accurately measured the overall level of mental attention in children with developmental coordination disorders clinically and was not significantly influenced by eye blinking.

## 3. Methodology

### 3.1. Materials

Neurosky’s mindwave is a device that measures brain activity using a sensor on the forehead (Fp1) and a clip located on the left ear that acts as a ground and reference. It can provide a raw signal at a sampling rate of 512 Hz and 12 bits of resolution as well as processed information like power bands δ, θ, α, β and γ, attention and meditation indicators. However, bands and indicators are sent at a rate of 1 Hz.

We also developed a training software, running on a tablet computer with an Android operative system and $10^{\prime \prime }$ screen, so that subjects could practice the control of their attention. Such software captures data conveyed from Neurosky’s sensor and stores them in an internal database. For neuro-feedback purposes, the whole screen shows a bar that moves up and down, changing its color according to the received attention values that ranged from 0 to 100 like a percentage.

**Figure 2:**
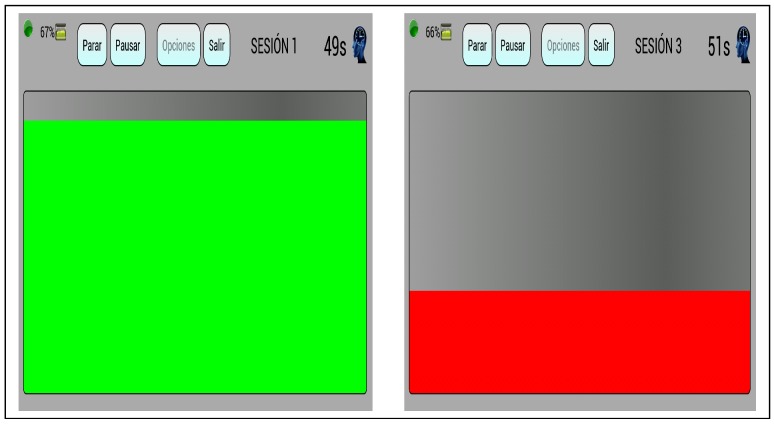
Screenshot during attention and non-attention trials. The left picture shows a big green bar associated to high values of attention. The right one shows a small red bar associated to low attention values.

The higher the attention value, the higher the bar shown on the screen (Figure 2). The color of such a bar is green for an attention level over 60%, red if it is under 40% and yellow otherwise.

### 3.2. Artifact Rejection

The software checks the POORSIGNAL indicator sent by Neurosky’s mindave every second. A value of 0 in this indicator guarantees good contact between electrodes and the skin and, therefore, a good quality signal. In the case of poor signal quality, the attention value is rejected and not recorded by the software.

On the other hand, the manufacturer also guarantees that the attention is obtained from applying an algorithm after removing the ambient noise and muscle movements from the raw brain signal [38]. Nevertheless, we included a second stage of verification of signal quality based on artifact detection in the non-overlapping epochs of 512 samples (1 s of duration) previous to the time the attention value is updated. If such an epoch does not contain muscle activity or blinks, then the attention value is admitted as a valid one.

**Figure 3:**
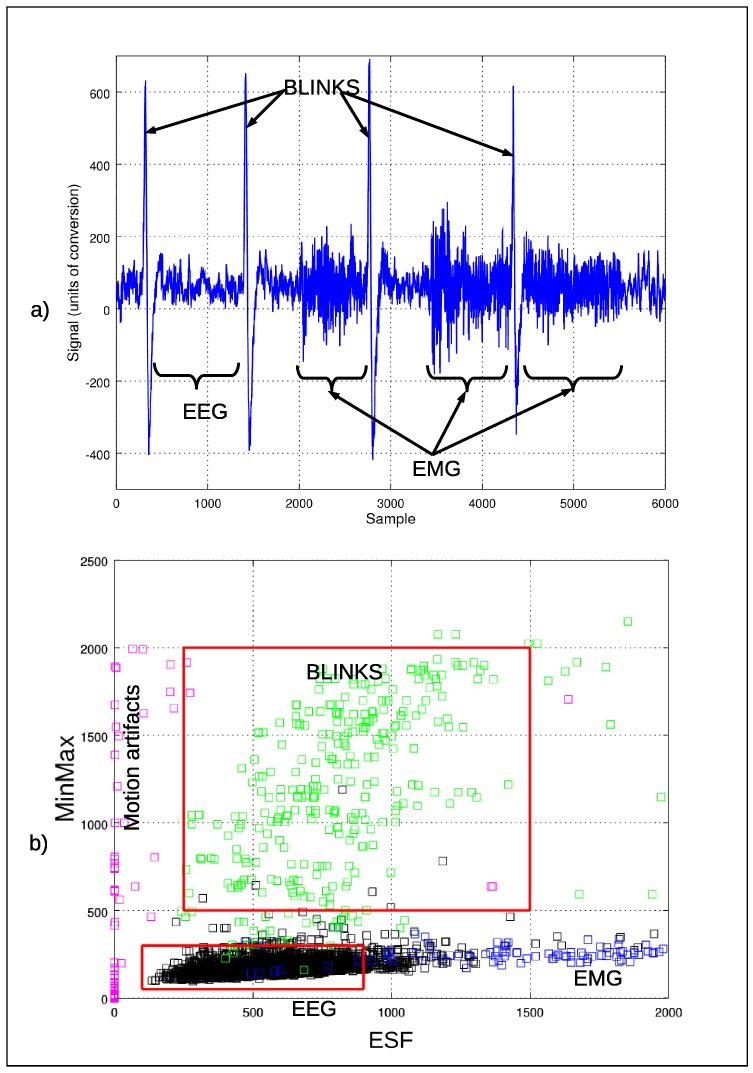
(**a**) a segment of a raw signal containing Electromyographic (EMG) artifacts and Blinks artifacts; (**b**) feature space wherein EEG signal epochs are in black, while epochs containing EMG, blink or motion artifacts are in blue, green and pink, respectively.

To accomplish this, we have used two features: the difference between the maximum and minimum sample value (MinMax), and the total energy (ESF) of the signal after applying a Savitzky–Golay lowpass filter (order 2 and length 35) [39] and substracting it to the raw signal. Figure 3 shows a segment of an EEG signal highly contaminated by EMG and Blinks artifacts and the obtained space of features. Epochs containing muscular activity have values of the MinMax feature that are similar or a bit higher than those of the epochs with only EEG, but with more energy from the filtered signal (ESF). Blinking or EEG-only windows have similar values in the ESF feature but differ in MinMax. Finally, windows with motion artifact contain values of these features that surround those obtained by other types of artifacts. For all of these reasons, the use of thresholds (maximum and minimum) of each dimension of the feature space has been proposed, to limit and facilitate the automatic detection of valid EEG container segments and blinks (as shown in Figure 3) with an accuracy of 96% and 98%, respectively. The method followed is conservative in the selection of valid epochs, reducing the number of false positives.

### 3.3. Participants

Seven normally developed subjects (A1, ⋯, A7) aged 36.4 ± 10.2 formed group A (control group) and three subjects with CP (B1, ⋯, B3) aged 35.3 ± 1.2 made up group B, who were recruited from ASPACE Sevilla, a non-governmental organization specialized in cerebral palsy. The recruitment into group B was done according to the following inclusion criteria:

1. The access to a computer by traditional switch-based devices is usually very hard to be carried out or almost impossible.
2. Have good intellectual capabilities.
3. Gross Motor Functional Classification System (GMFCS) Level V [40].
4. Communication Function Classification System (CFCS) Level IV [41].

Although ASPACE is the most important association dedicated to deal with people with CP in the province of Seville, there were not many people who met the inclusion criteria, so it was difficult to perform the experimentation with a large population. Only six out of 69 subjects met it, but just three of them took part in this experiment.

The participants agreed to take part in the experiment and in the case of group B, their families were informed and allowed their participation. The Ethics Committee of the University of Seville also approved this experiment.

### 3.4. Conditions

Experimentation was carried out in a quiet room with dim lighting. The experiment was considered correct if there were no interruptions. Participants belonging to group A were told to set the environmental conditions (temperature, lighting) so that they were comfortable during the experiment. For group B subjects, experimentation was conducted by a caregiver who was always present and set the environmental conditions.

### 3.5. Phases in Experimentation

**Figure 4:**
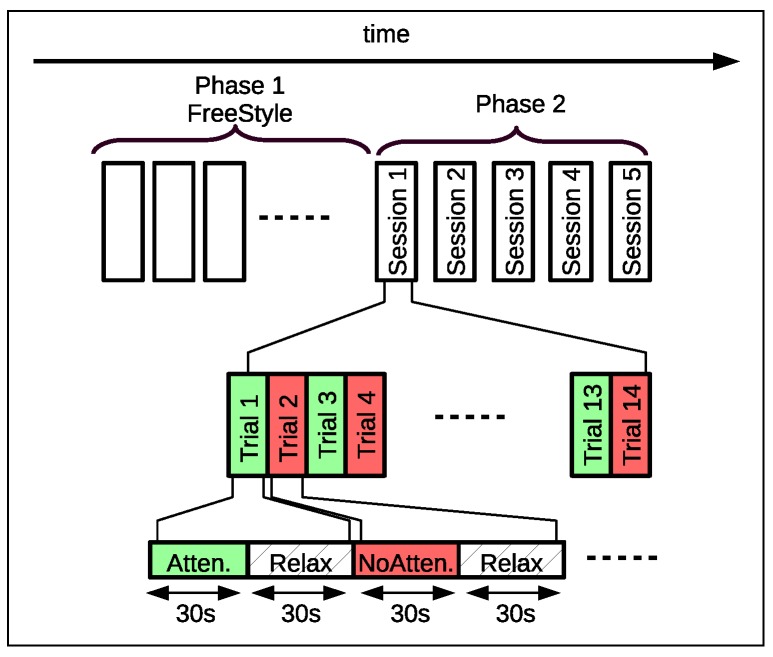
Experimental time sequence. Phase 1: Subjects must find the strategies to control their attention levels. A maximum of ten 15-min sessions was set. Phase 2: Five 14-min sessions with seven attention/non-attention trials. Each trial contains an ending relaxing period of 30 s.

Experimentation consisted of two phases (see Figure 4). As explained below, in the first phase, the participants had to find the strategies to control their attention. Those who would not have been able to control their mental state properly did not perform the following phase. The second phase was similar to the first with the difference that we recorded the information sent by the sensor during the attention/non-attention trials.

#### 3.5.1. Phase 1

The main goal of phase 1, also called ”Freestyle”, was to practice and try to find the best strategies to control attention levels. Previously, they were told to follow a series of basic strategies. For instance, to practice attention we told them: ”try to perform mathematical operations”, ”try to plot an object mentally”, etc. To practice non-attention, we suggested: ”try not to think about anything”, ”make your mind go blank”, etc. These suggestions were to get them going, and they each had to find the best way of controlling her/his level of attention. We used the software explained above to give participants feedback about how they were performing the experimentation. The caregiver sometimes asked participants in group B to perform several attention/non-attention actions to get some feedback about their achievements.

The number of sessions in phase 1 depended on the subject, but to prevent this phase from becoming too drawn out, we set an upper limit of 10, roughly 15 min sessions.

At the end of each session in this phase, group A participants were asked to fill in a short questionnaire about how well they had performed the experiment. Those who admitted not having controlled attention properly in more than two out of the last five sessions were excluded from the following phase. In group B, the caregiver was responsible for discriminating such participants.

#### 3.5.2. Phase 2

In this phase, participants performed a sequence of 5, 14-min sessions (one per day). Each session consisted of 14, 1-min, trials divided into two 30-s parts. In the first part, subjects had to keep their attention level above or below a threshold of 50% as soon as the application requested it. In the last 30-s part of the trial, the subject had to relax and, to help participants do so, the software showed an idyllic landscape on screen. Attention/non-attention experiments were made in odd and even trials, respectively. Figure 4 shows the time schedule of this phase.

The software recorded the processed information sent by Neurosky (attention level, power bands, etc., see Section 3 for more details). For each participant and session, a total of 10 parameters/s × 14-min × 60 s/min were obtained for posterior analysis. As in the previous phase, a three-question survey (Table 1) was given to participants to be answered using a three Likert item rating as follows: 1 (no, badly), 2 (neutral), 3 (yes, well). Obviously, for group B, the survey was filled in by the caregiver after asking and interpreting subjects’ answers. The difficulty in interpreting subjects’ answers was the main reason to select such a reduced number of responses on the Likert’s scale.

## 4. Results

Data were analyzed using GNU Octave version 3.8.1 and R version 3.0.2. The first analysis was to find out how the method for identifying attentional states had worked. As the variable selected to control feedback to the user was the attention signal, the exploratory analysis was based solely on this. Furthermore, we will look at other signals in the study at a later stage.

Phase 1 removed four participants from group A and one for group B. Namely, participants A5–A7 and B1 were unable to control their attention level and did not go on the following phase.

### 4.1. Exploratory Analysis

**Figure 5:**
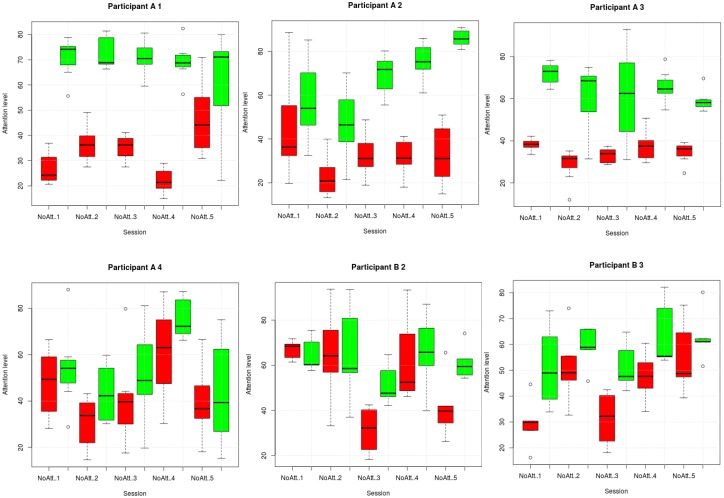
Attention levels for participants and sessions. Green boxes contain averaged values for attention trials; red boxes the averaged values for non-attention trials.

Figure 5 shows boxplots containing the results of phase 2 for each subject and session, differentiating between attention trials (green boxes) and non-attention ones (red boxes). Each box contains seven values representing the average of the attention percentages of a trial in a session. The figure shows that subjects A1, A2 and A3 performed the experiment rather well, as the attention boxes generally contained higher values (above the 50% threshold) than the non-attention ones (below 50%) and there was not excessive overlapping among them. It was clearly not easy to perform all sessions of the experiment perfectly. For example, participant A1 did not obtain good results in the last session; neither did A2 in the first and second sessions nor A3 mainly in the attention trials in session 3. Participants A4 and B3 behaved differently; they did not fulfill the goals since many of their results in the attention trials were below the threshold and many of those in the non-attention trials were above it. However, we should remark that for these two subjects in each session, the median values in the attention trials were higher than in the non-attention ones. Participant B2 performed similarly to A4 and B3 in the last three sessions. In the others, the subject’s attention level was almost always above the threshold with non-attention mean values higher than those in attention trials.

Table 2 shows the mean and standard errors of some quantitative features that may characterize experimental results:

- Successful score (SS). Percentage of time the subject met the goals: that is, when the attention level was kept above the threshold of 50% in attention trials or below it in non-attention ones.
- The initial time, ${\bar{t}}_{i}$ or time elapsed, on average, from the beginning of the trial until the subject made the attention level go above/below the threshold in attention/non-attention trials, respectively. We can differentiate ${\bar{t}}_{i}$ for attention and non-attention trials calling it $t_{on}$ and $t_{off}$, respectively.
- Sustained attention time, ${\bar{t}}_{s}$, shows how long, on average, the subject could maintain the attention level without crossing the threshold.

Successful score and sustained time are fulfillment indicators of the experiment and they are dependent to a certain extent; thus, as SS increases, so does the sustained time. People who obtained high SS values in both types of trials performed the experiment better than those who obtained lower SS values (close to 50%) or unbalanced results between trials. Sustained time, *${\bar{t}}_{s}$*, is strongly affected by the number of threshold crossings. Therefore, further away from the threshold, the attention level produced by the subject, the higher the value of the sustained time. A participant producing an attention level close to the threshold value is more likely to cross it and obtain a lower sustained time.

According to these parameters, in group A, participants A1, A2, A3 performed the experiments quite well, since their successful scores were high and balanced between attention and non-attention trials. The sustained times were, in general, long (greater than 16.1 s) for them, although participant A3 obtained a lower result in non-attention trials. Participant A4 found it difficult to keep the attention level above/below the threshold so the SS values and the sustained time were the lowest achieved by participants in group A.

In group B, participant B2 obtained unbalanced percentages between trials, which meant it was difficult for him to maintain the non-attention state for long. The sustained time for this participant also confirms this fact. Subject B3 was able to control the two states in a balanced way but not for long, as the sustained time indicates. In general, participants in group B performed worse than the other participants.

**Figure 6:**
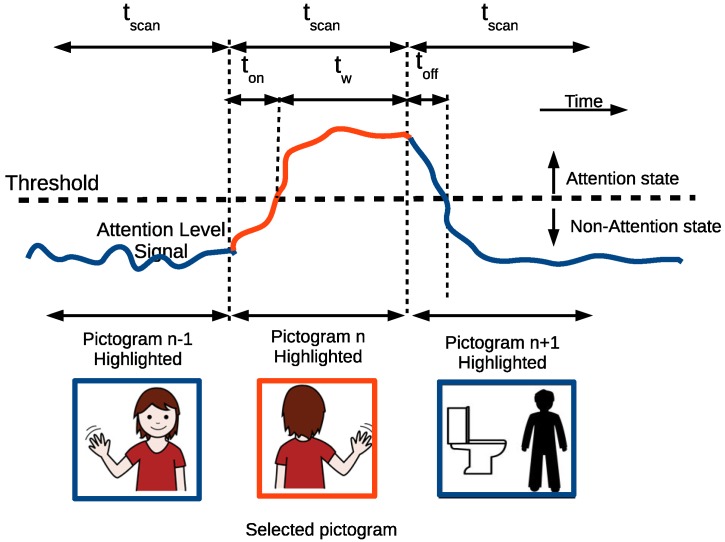
Temporal parameters and their relationship with the scanning period. $t_{scan}\geq t_{w}+t_{on}$ to select one pictogram and $t_{w}$ also has to be greater than $t_{off}$ ($t_{w}>t_{off}$) so as not to select the following pictogram.

Initial time ${\bar{t}}_{i}$ and sustained time ${\bar{t}}_{s}$ are related to the time needed to select a pictogram on a communicator board, when accessing a computer by changing the attentional state. Firstly, a threshold establishes the border between these two states, so a subject who wants to select a pictogram has to exceed such a threshold for a time. The time *${\bar{t}}_{i}$* in attention trials ($t_{on}$) shows the average time to cross such a threshold and reach the attention state. In the same way, the time ${\bar{t}}_{i}$ in non-attention trials ($t_{off}$) shows the time taken to go back to the non-attention state. In between them, the attention level must be kept high for $t_{w}$ seconds so that the system can detect the user’s intention (see Figure 6). The dwell time or scanning period $t_{scan}$ depends on such temporal parameters. For example, participant A1 took $t_{on}$ = 2.48 s to change from ’resting’ to the attentional state and $t_{off}$ = 2.08 s to come back again. This means that the scanning period, $t_{scan}$ has to be greater than 2.48 s (Equation (1)) on average and the $t_{w}$ greater than 2.08 s to avoid selecting the pictogram next to the preselected one (Equation (2)). The selection time, $t_{w}$, is also related to sustained time, ${\bar{t}}_{s}$, as the latter sets the upper limit for the former. Table 2 shows that all participants were not able to maintain their attention state for more than 10.7 s in group A or 11 s in group B:

(1) $$\begin{array}{c} t_{scan}\geq t_{on}+t_{w}, \end{array}$$

(2) $$\begin{array}{c} t_{off}<t_{w}\leq {\bar{t}}_{s}. \end{array}$$

In Section 5, we study the optimal $t_{w}$ and the performances of detection of the attentional state.

### 4.2. Test Results

The results of the survey are shown in Table 3.

Group A participants did not feel tired during experimentation (1.5), but those in group B did feel tired after finishing the experimentation (2.7).

Quantitative data and the results of the survey for group A shows agreement between them. For example, participants A1, A2 and A3 said they found it relatively easy to keep their attention level high (2.65), although it was easier to keep a low attention level (2.9). Only participant A4 rated these questions lower than the others. Group B participants thought they kept their attention level high (3) or low (2.7) very well, although this did not concur with their results.

### 4.3. Effect of High/Low Levels of Attention on the Power Bands

In line with the latest research, we first investigated the significance level of δ, θ, α, β and θ/β bands using the Wilcoxon rank-sum test analysis applied to each subject individually. Data were analyzed trial by trial to filter out outliers. We did this by estimating the interquartile range (IQR). Values out of bounds [Q1 − 1.5 × IQR, Q3 + 1.5 × IQR]—where Q1, Q3 were the first and third quartile, respectively—were considered outliers and ignored in the calculation of the average attention level and power bands for each trial and session. Finally, we averaged the attention level and power bands for each trial and subject, so we used a number of 5 × 7 × 2 = 70 (number of sessions x number of trials of a type x type of trial) for the statistical analysis. Table 4 shows the *p*-values obtained by the Mann–Whitney–Wilcoxon test.

The attention level was significant between type of trials for most of the participants, and it was the best indicator, followed by the θ/β ratio, which showed significant differences in four out of six subjects. The remaining power bands had significant differences in three out of six participants.

Our results showed that, during the attention trials, the δ power and the ratio θ/β decreased, whereas the γ power increased. The rest of the power bands had a non-homogeneous behavior among participants. For example, α and θ bands diminished during attention trials for two out of three participants, but they had the opposite effect on the other subject. Table 5 summarizes the effect that an increment in attention had on power bands for each participant. Only the cells associated with statistically significant differences between sorts of trials were filled in.

The fact that the δ band was lower in attention trials does not match with literature [32,42], which has reported an increase in this band mainly in frontal leads in different tasks: mental calculation, semantic tasks, and the Sternberg paradigm. The explanation is that one of the strategies our participants followed to be inattentive was to look at different parts of the room. This helped them to avoid focusing on an idea, thought or object, but the ocular movement might have interfered in this band. An increment in γ band in attention has also been reported in several studies. In [43], a model is presented where sustained attention relies on frontomedial θ oscillations and selective excitation and inhibition of cognitive processing through γ and α oscillations, respectively. The study in [44] shows how spatial attention increases high-frequency γ synchronization in human medial visual cortex. Paying attention to a sustained tactile stimulus amplifies contralateral γ oscillations as reported in [45]. Finally, a reduced $\frac{\theta }{\beta }$ during attention trials is also coherent with scientific literature related to ADD or ADHD [33].

### 4.4. Classification Analysis

Statistical analysis suggests that it might be worth including more information, such as the power bands, to improve discrimination in the level of attention, and that is covered in this section. Firstly, we studied the whole data length contained in each trial in the classification analysis; we then looked at the effect of shortening such data lengths in Section 5.

Each trial contains a set of 30 × 11 data comprising 30-s windows of nine power bands, attention and meditation values (the last one was not included in this analysis). First of all, outliers were filtered out from each power band following the same procedure explained in the previous section. After this, each band and the attention percentages were averaged, reducing the original amount of data in a trial down to only 10 values.

This two-class classification analysis was based on three different sets of features. The first one, or Set1, used the averaged attention for each trial and session; thus, it contained a total of 5 × 14 features. The second set, or Set2, included attention and the θ/β band, so 5 × 14 × 2 features were used. Finally, Set3 contained almost all the information the sensor sends, hence there were up to 5 × 14 × 10 features.

**Figure 7:**
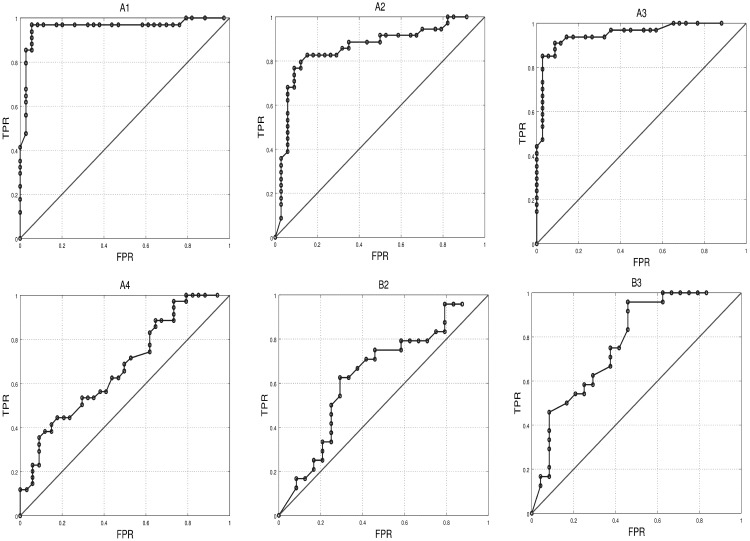
Mean Receiver Operating Characteristic Curves (ROC) for K-fold cross-validation method using the set of features with highest AUC value.

Classification was based on LDA (linear discriminant analysis) and a 4-fold cross-validation method. Table 6 shows the results obtained for two indicators of classification performance: accuracy and AUC (area under ROC curve—shown in Figure 7). According to accuracy, it is better to use Set1 to train the classifier because most of the participants obtained better results with it (four out of six). According to AUC, both Set1 and Set3 shared the same number of successful participants. We can see from ROC curves that the classification was fair for A4 and B2 with AUC ∈ [0.5 0.75], good for A2 and B3 (AUC ∈ [0.75 0.92] and very good for the rest (AUC ∈ [0.92 0.97]).

## 5. Accessing a Communication Board by Modulation of Attention: Estimation of Performances

Classification results showed in the section above were based on a set of features obtained by averaging 30-s data windows in each session. In a more realistic situation, where attention might be used by a user to access a computer, it does not seem very useful either to wait 30 s for the system to estimate the attentional state, or use 30-s trials to train the classifier. Shorter data lengths should be chosen to speed up the communication rate. In this section, we study the classification performances in a more realistic situation, but based on the data we collected in experimentation.

### 5.1. Temporal Parameters and Their Relationships

**Figure 8:**
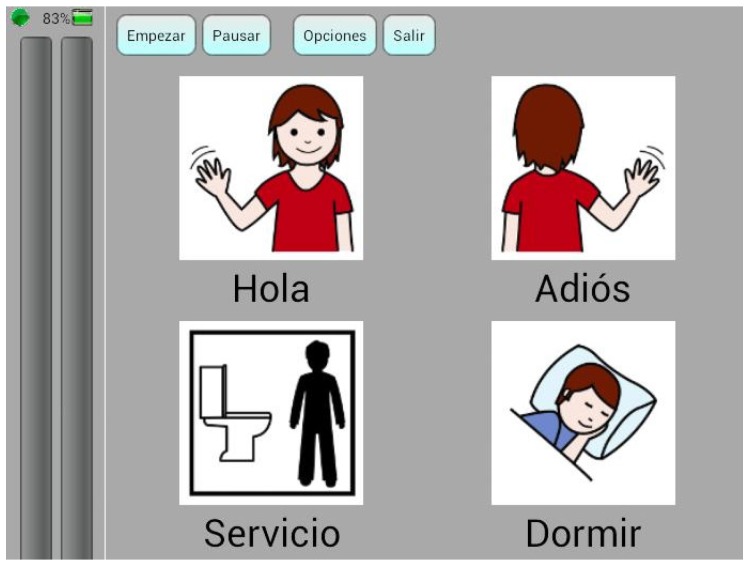
Communication screen. Captions read from top, left to right: Hello, Goodbye, Toilet, Sleep.

As explained in detail in Section 4.1, the communication board (see Figure 8) would be operated in a similar way to a switch, requiring a linear scanning of its pictograms [47]. A user must keep the attention level low most of the time the scanning is running and increase it very quickly when the desired pictogram is highlighted. At his point, the user has to raise the attention level over a threshold for a period of time, $t_{w}$. Finally, the attention level has to drop again to prevent the selection of the following pictogram. Figure 6 shows the temporal parameters involved in this mode of operation: $t_{on}$, $t_{w}$, $t_{off}$ and $t_{scan}$. The sum of the first two parameters should be less than the scan period, $t_{scan}$ (Equation (2)).

For a fast communication rate, $t_{scan}$ should be as short as possible, and therefore $t_{w}$. We estimated $t_{on}$ from attention trials in a similar way to what we did before, but taking into account that, in this case, the threshold is set by the classifier instead of being arbitrarily fixed at 50%. Following the same procedure, but with non-attention trials, we estimated the ’disconnection’ time $t_{off}$. The time or length of data used to train the classifier and test it, $t_{w}$, is variable in this new analysis, changing from 2 s up to 15 s. The reason for not using a higher upper limit lies in the fact that the average sustain time, ${\bar{t}}_{s}$, for all participants was around 14 s (see Table 2). Therefore, instead of using 30 s of data for training, we used only the first $t_{w}$ seconds.

A final issue concerns the user’s ability to maintain the non-attention state. We know from experimentation that the attention level is not kept under the threshold for long with several threshold crossings occurring during a trial. Therefore, there were intervals of time (pulses), of a variable duration, in which the attention level was higher than the threshold. These pulses could select unwanted pictograms. To reduce these false positives in non-attention trials, we studied the average number of pulses, $N_{p}\left(t_{w}\right)$, of length greater than or equal to $t_{w}$ that there were in the $T_{non-att}$ = 30 s of the trial. Let us suppose a communication board with $N_{icons}$ in which only one of them has to be selected. The user has to keep the attention level low for $\left(N_{icons}-1\right)\times t_{scan}$ seconds and high for $t_{w}$ seconds when the desired icon is highlighted. To avoid false positives during the low attention period, time $\left(N_{icons}-1\right)\times t_{scan}$ must be lower than approximately the ratio $\frac{T_{non-att}}{N_{p}}$ that establishes the upper limit (Equation (5)). This is an optimistic estimation of such a limit because the duration of the whole trial (30 s) was split among the number of pulses it contained and the pulses’ duration itself was not substracted from the 30s.

Assuming that $t_{on}$/$t_{off}$ follows a normal distribution, we can modify Equations (1) and (2) to include the variability of these temporal parameters. Let $\sigma_{t_{on}}$ / $\sigma_{t_{off}}$ be such a variability. Then, Equation (3) guarantees that $t_{scan}$ contains the 95% of $t_{on}$ values, leaves $t_{w}$ seconds for the classifier to detect the attentional states and, therefore, maximizes the number of true positives. In the same sense, Equation (4) guarantees that the window size $t_{w}$ is greater than the 95% of $t_{off}$ values and minimizes the number of false positives:

(3) $$\begin{array}{c} t_{scan}=\left({\bar{t}}_{on}+1.64\times \sigma_{t_{on}}\right)+t_{w}, \end{array}$$

(4) $$\begin{array}{c} t_{w}>{\bar{t}}_{off}+1.64\times \sigma_{t_{off}}, \end{array}$$

(5) $$\begin{array}{c} N_{p}\times \left(N_{icons}-1\right)\times t_{scan}<T_{non-att}. \end{array}$$

The optimal $t_{w}$ is the minimum value of $t_{w}$ that satisfies Equation (3)–(5). The procedure depicted in Algorithm 1 explains how the optimal $t_{w}$ and accuracy were obtained.

**Algorithm 1** Procedure for estimating optimal window length and accuracy.
1:**for** $t_{w}$ = 2 to 15 **do**2: Estimate ${\bar{t}}_{on}$, $\sigma_{t_{on}}$ ${\bar{t}}_{off}$, $\sigma_{t_{off}}$ Accuracy and $N_{p}$3: $t_{scan}={\bar{t}}_{on}+1.64\times \sigma_{t_{on}}+t_{w}$4: **if** $N_{p}\times \left(N_{icons}-1\right)\times t_{scan}<T_{non-att}$ AND $t_{w}>\;(t_{off}+1.64\times \sigma_{t_{off}}$) **then**5:  Optimal $t_{w}^{opt}$ = $t_{w}$6:  Accuracy($t_{w}^{opt}$) = Accuracy7:  Stop8: **end if**9:
**end for**



### 5.2. Classification Analysis

As sessions took place on different days and there was a certain variation in data from session to session (see, for example, the attention values in Figure 5), we thought it better to focus on the individual performances of the classifier in each session and then average the results of the five sessions. This means that data for training, validation and test sets were extracted from each session. The accuracy, $N_{p}$ and temporal parameters $t_{on}$ and $t_{off}$ were obtained from them, repeated for the rest of the sessions and averaged. Six out of fourteen trials of each session were randomly selected for training, four for validation and the other four for testing. These data sets were well balanced between the attention and non-attention trials they contained. The validation data set determined the optimal data lengths for the classifier whereas the test set assessed the performances for such an optimal length. This procedure was repeated ten times for each session, changing the trials which belonged to each set and then averaging the results.

For the *training set*, features were obtained in a similar way to that was explained at the beginning of Section 4.4 apart from the length of the window. A total of 3 × 2, 3 × 2 × 2, 3 × 2 × 10 features were used for training based on Set1, Set2 and Set3, respectively.

We adopted a slightly different approach to extract features for the validation and test sets. In thiscase, instead of selecting the data from the beginning of the trials, we looked for the first projected value which exceeded the threshold set by the classifier. This value marked the starting position of the data window used to obtain the features. This approach allowed us to obtain better results than if we had followed the same procedure as for the training set. A total of 2 × 2, 2 × 2 × 2, 2 × 2 × 10 features were used for validation and testing based on Set1, Set2 and Set3, respectively.

**Figure 9:**
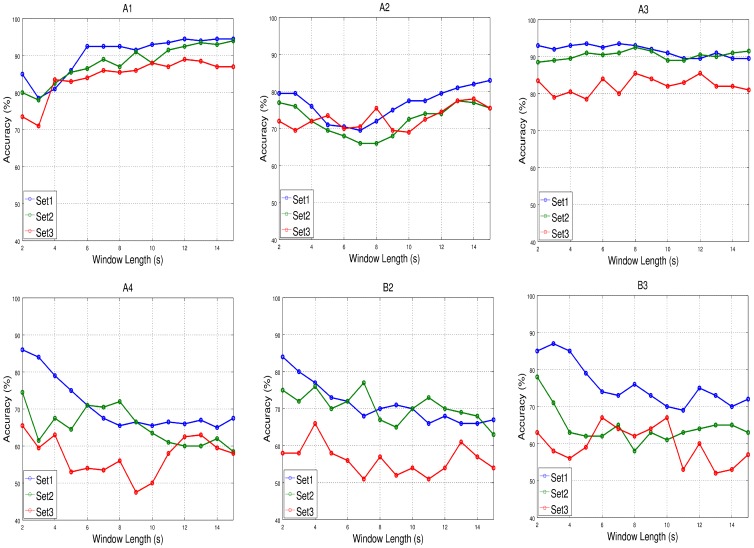
Accuracy against window length, $t_{w}$, for each participant and set of features. For most participants, the highest accuracies were obtained using the Set1 of features and short window lengths.

**Figure 10:**
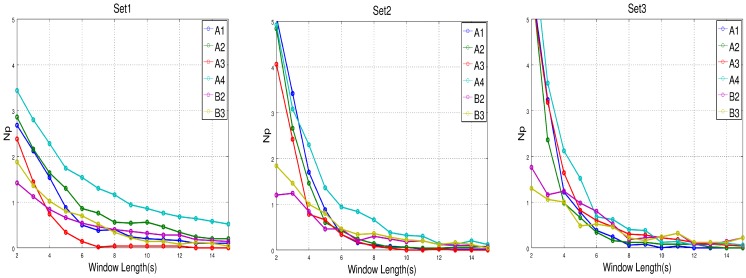
Average $N_{p}$ against $t_{w}$ for each participant and set of features. As expected, $N_{p}$ decreases as $t_{w}$ increases. Optimal $t_{w}$ must be chosen to reduce $N_{p}$, increase the accuracy and minimize $t_{scan}$ .

Figure 9 shows the relationship between the averaged accuracy and the length of the window, $t_{w}$, for each participant and set of features based on the validation set and Figure 10 the averaged $N_{p}$. We can see in Figure 9 that accuracy increases for A1 and A2 and decreases for the rest of the participants as the length of the window used to obtained the features increases. Table 6 shows that only A1 and A2 could keep the attention high/low on average for more than 14 s (the maximum length of the window used in this analysis). According to this, A3, A4, B2 and B3 would obtain worse results because longer data windows are more likely to contain attentional values that do not belong to the correct class. Therefore, averaging those values to get the features for the two classes would make them closer and reduce the performance of the classifier. In general, the tendency is that the accuracy decreases as the length of data used to train the classifier increases for subjects who could not maintain their attentional state for too long. Nevertheless, for the rest of participants, the effect might be to maintain the accuracy or slightly improve it as the length of data increases.

It seems that small window lengths benefited the accuracy for the Set1 classifier, but it worsened $N_{p}$ values, which means that the probability of making false positives increases. Table 7 presents the optimal results for each set of features and participant and a 4-pictogram communication board. The most accurate results and their associated window lengths are in bold. For most of the participants, the best set of features was the one based on Set1. The asterisk in participant A4 and Set1 shows that the procedure to obtain the optimal window did not find any one that satisfied Equations (3)–(5).

Table 7 shows that the average accuracy for Set1 was 84.4%, which improves the accuracy of 76.4% obtained in [48] where the attention level of healthy students was measured using the same sensor as in our research. In that study, students performed foreign language exercises and a support vector machine was used as a classifier.

Table 7 shows that the time needed to select one pictogram and not the rest of them on a 4-icon communication board must be equal to or greater than 4 s and with a $t_{scan}$ around 10 s. For example, participant A3 would need a $t_{scan}$ > 10.6 s (4 + 3.3 + 1.64 × 2) and 42.37 s for the scanning to move throughout the four icons. An increase in the number of icons would probably require higher $t_{w}$ values. This could imply a worsening in accuracy for some participants (for example A4, B2 and B3, whose accuracy curves showed a negative dependency on $t_{w}$). Other participants had high accuracy for a wide range of $t_{w}$ values, so their accuracies would be relatively unaffected by this fact.

### 5.3. Information Transfer Rate

The information transfer rate (ITR) [49,50] depends on the number of choices or thoughts (two in our case: attention high/low), the accuracy and the time the classifier needs to make a prediction. Figure 9 shows that for the shortest window length used for classification, $t_{w}$ = 2 s, the accuracy is roughly over 80% for all participants when Set1 is used. Therefore, the participants in group A obtained an averaged ITR of 12.74 bits/min while the others had 11.34 bits/min.

### 5.4. Comparison with BCI Systems

According to [2], these values of ITR would place this BCI method with others like those based on slow cortical potential (SCP), with an ITR between 5–12 bits/min, and sensorimotor rhythms (SMR), with an ITR between 3–35 bits/min, and approaching others such as the 14.4 bits/min obtained for healthy people using a P300 system [51]. However, these results are far away from other systems, such as steady-state visual evoked potentials (SSVEP). For example, in [52], the authors obtained an ITR that was equal to 105 bits/min.

Other BCI interfaces are based on the hemodynamic response instead of the firing activity of the cortical neurons. Near Infrared Spectroscopy (NIRS) is a relatively new measurement modality [53] that measures such a response. As the hemodynamic is temporally delayed from the onset of the underlying neural activity, it is expected for a latency of several seconds to appear in response to a change in participants’ behavioral or mental states. This might severely limit the practical use of such systems. BCIs based on NRIS try to classify subjects’ intentions during motor imagery [54,55], arithmetic tasks [56,57,58] or both [59]. Typical ITRs for these systems are below the 4 bits/min [2] with accuracies ranging between 65–96.3% among them. Nevertheless, in a recent study [60], a complex NIRS device with 30 channels and a four-class classifier obtained an ITR equal to 5.82 bits/min with an accuracy of 87.58%. This result outperforms previous NIRS systems, but it is still far from the upper limit of our proposal.

### 5.5. Comparison with Non-BCI Systems

For people with severe disabilities, a system that reliably allows them to access a computer as fast as possible and with low effort would be highly beneficial. For example, in late stages of ALS, some people can control their eye movements. This lets them use several interface techniques such as eye tracking interfaces (ETI), BCI or Blink/Wink detection systems. A blink or a wink detection can be carried out by both measuring the electrical activity of the muscles controlling the eyelids or through image processing.

A comparison between ETI and P300 interfaces was accomplished in [61]. Results showed that ITR and usability of the eye tracker were higher than the P300. Moreover, the cognitive workload was higher for the BCI. A more recent study [62] showed that an SSVEP BCI obtained results comparable to the ETI in terms of accuracy and ITR. In particular, when the size of a target was relatively small, the BCI had significantly better performance than the ETI. In [63], an image processing system is described to detect voluntary winks with an accuracy of of 95% for temporal windows of 2 s, which makes an ITR equal to 21.5 bits/min, in the range of P300 BCI systems [2]. According to this information, only SSVEP systems can outperform the interfaces based on eye tracking or blink/wink detection.

Nevertheless, not all people with severe disabilities can control their eyes. For example, some people with ALS or with locked-in syndrome (LIS) can not move the eye accurately or simply open/close their eyelids. For them, the ETI or the blink/wink detection systems are not suitable, In [64], the authors evaluated electrooculography, an eye tracker and an auditory brain-computer interface as access methods for an LIS subject. He was able to communicate with slow residual eye movements and rated the ease of use of the BCI as the highest among the tested systems, because no precise eye movements were required, but also as the most tiring. When no eye control is possible, an eye-gaze independent BCI system could get into action. We can classify these BCI systems according to the stimulus: tactile, auditory or visual. The latter is a BCI system that does not require the subject to look at different parts of the screen. All information appears very close and around a central point or sequentially in the same position. In [65], there is a comparison among such systems. The faster auditory BCI that the authors found has an accuracy of 94% and an ITR equal to 10 bits/min, better than tactile and similar to visual BCI but based on the rapid serial visual presentation paradigm (RSVP). Our proposal could easily be adapted as a gaze independent BCI depending on whether the pictograms are shown sequentially in the same place.

To sum up, the modulation of attention can be used as a gaze independent BCI for people with residual eye movement with slightly higher ITR than other counterpart BCIs and outperforms other techniques that are not applicable there.

### 5.6. Training Time

SCP- or SMR-based BCIs require long training times in comparison to the method of modulating the attention. In this study, we used up to 150 min (10 session of 15 min) of training, although some participants achieved the required skills in a few sessions. In [66], a range between 240–320 min of training was carried out to train an SMR BCI system. Longer periods of training have been reported for SCP-based systems. For example, in [67], two people with ALS were trained for more than one year.

## 6. Discussion

This study shows that not all participants were able to manage their attentional state well enough. Three participants from group A and one from B were not able to start the phase 2 (40%) after 150 min of training.

An overall amount of 60% of the initial participants turned out to be potential users of this technology by achieving an accuracy of over 70%, which is the threshold proposed in [68]. It is well known that not everybody can control a BCI system, being known as “BCI illiterates”. According to [69,70], about 20% of subjects are not proficient with a typical BCI system. This percentage was reduced down to 3.59% in SSVEP BCI [71] where 53 people took part in only four sessions. Therefore, the percentage of “illiterates” obtained in this experiment was large compared to the normal percentages in other sorts of brain computer interactions. Nevertheless, a study with healthy people controlling a BCI system by means of SCP [72] reported that, after 180 min of training, the percentage of people who could achieve significant voluntary control of brain signals was 66%, similar to the one obtained in this study.

In group B, participants achieved the skills with an accuracy of 76%. However, it is also remarkable how tired this method of access made people with disabilities, scoring it an average 2.7 out of 3. For group A, all participants achieved the skills with an average accuracy of 82.4%, and they did not get tired during the experimentation (1.5).

For group A participants, the average $t_{scan}$ was equal to 10.95 s, whereas for group B, it was similar at 10.16 s A higher number of icons in the communicator means increasing the $t_{w}$ (which diminishes $N_{p}$) to reduce the number of errors committed during the non-attention phase. Thus, the communication board should not contain too many icons (estimated between four and seven). This methodology has an ITR close to other classical BCI methods such as SCP and SMR but lower in comparison with others based on P300 or SSVEP and non-BCI such us ETI or wink detection systems. Moreover, we can use it as an independent eye BCI with similar results as other counterparts BCI.

There were significant differences in attention values between types of trials for most of the participants, whereas the θ/β ratio was also significant in four out of six participants and other power bands were significant in three out of six participants. Our study reveals that the δ power band and θ/β ratio decreased in attention trials in comparison with non-attention ones, since the γ power band had the opposite behavior, increasing during attention trials and decreasing in the other trials. Including power bands into the classifier did not improve the accuracy in discriminating the two attentional states. The best results were obtained by using only the attention indicator in classification.

## 7. Conclusions

This study has presented some findings about using the modulation of attention as an input method for people with disabilities. However, further research must be carried out including the communicator in the experimentation, with a larger sample of subjects with disabilities (even other collectives such as people with ALS) and other kinds of classifiers.

## Figures and Tables

**Table 1:** Test questions to be answered at the end of each session in phase 2.

(a) Could you keep your attention level high when required?
(b) Could you keep your attention level low when required?
(c) Did you get tired?

**Table 2:** Successful score, initial time and sustained attention time for each participant. Standard errors (SE) are also shown.

Subject	Condition	SS (%)	${\bar{t}}_{i}\pm \mathrm{SE}$ (s)	${\bar{t}}_{s}\pm \mathrm{SE}$ (s)
A1	Attention	86.0	2.48 ± 0.87	19.3 ± 3.7
	Non-attention	85.4	2.08 ± 1.02	18.6 ± 5.8
A2	Attention	79.1	4.29 ± 1.44	18.8 ± 4
	Non-attention	83.6	2.19 ± 0.62	17.7 ± 1.9
A3	Attention	80.8	2.06 ± 0.30	16.1 ± 1.5
	Non-attention	86.0	1.69 ± 0.19	12.5 ± 0.8
A4	Attention	57.9	2.91 ± 0.66	10.7 ± 2.5
	Non-attention	63.1	4.63± 1.67	11.4± 2.0
B2	Attention	71.9	2.2 ± 0.60	12.4 ± 1.8
	Non-attention	46.8	5.0 ± 2.14	7.8 ± 2.8
B3	Attention	69.5	2.0 ± 0.46	11.0 ± 1.3
	Non-attention	63.7	2.6 ± 0.62	11.0 ± 2.5

**Table 3:** Results of the survey: (a) keep attention high; (b) keep attention low; (c) tiredness. Participants had to rate each question as follows: 1 (no, badly), 2 (neutral), 3 (yes, well) at the end of each session in phase 2. Each cell contains the averaged rating among sessions.

Participant	(a)	(b)	(c)
A1	2.8	3	1
A2	3	3	2
A3	3	3	2
A4	1.8	2.6	1
Mean	2.65	2.9	1.5
B2	3	2.6	2.6
B3	3	2.8	2.8
Mean	3	2.7	2.7

**Table 4:** *p*-values obtained by Mann–Whitney–Wilcoxon test.

Subject	Attention	δ	θ	α	β	γ	θ/β
A1	**<0.001**	**<0.001**	**<0.001**	**<0.001**	**<0.001**	0.02	**< 0.001**
A2	**<0.001**	0.12	**<0.001**	**<0.001**	**<0.001**	**<0.001**	**<0.001**
A3	**<0.001**	**<0.001**	**<0.001**	**<0.001**	0.43	**0.02**	**<0.001**
A4	**0.04**	0.47	0.79	0.92	0.34	0.56	**0.04**
B2	0.15	0.32	0.87	0.48	0.31	0.30	0.52
B3	**<0.001**	**<0.001**	0.65	0.12	**0.04**	0.06	0.43

**Table 5:** Attention effect on power bands between type of trials. Subscripts a and n denote attention and non-attention trials, respectively.

Band	A1	A2	A3	A4	B3
δ	$\delta_{a}<\delta_{n}$	-	$\delta_{a}<\delta_{n}$	-	$\delta_{a}<\delta_{n}$
θ	$\theta_{a}<\theta_{n}$	$\theta_{a}>\theta_{n}$	$\theta_{a}<\theta_{n}$		-
α	$\alpha_{a}<\alpha_{n}$	$\alpha_{a}>\alpha_{n}$	$\alpha_{a}<\alpha_{n}$	-	-
β	$\beta_{a}<\beta_{n}$	$\beta_{a}>\beta_{n}$	-	-	$\beta_{a}>\beta_{n}$
γ	$\gamma_{a}>\gamma_{n}$	$\gamma_{a}>\gamma_{n}$	$\gamma_{a}>\gamma_{n}$	-	-
θ/β	${\frac{\theta }{\beta }}_{a}<{\frac{\theta }{\beta }}_{n}$	${\frac{\theta }{\beta }}_{a}<{\frac{\theta }{\beta }}_{n}$	${\frac{\theta }{\beta }}_{a}<{\frac{\theta }{\beta }}_{n}$	${\frac{\theta }{\beta }}_{a}<{\frac{\theta }{\beta }}_{n}$	-

**Table 6:** Classification results according to the set of features. Accuracy is given in percentage while AUC is a dimensionless quantity. A fair or a good classification result is obtained for AUC values ranged between [0.5 0.75] or [0.75 1], respectively [46].

Subject	Vble	Set1	Set2	Set3
A1	Accuracy (%)	**95.59**	94.12	91.18
	AUC	**0.965**	0.958	0.962
A2	Accuracy (%)	**80.88**	75.00	70.58
	AUC	**0.856**	0.819	0.776
A3	Accuracy (%)	**92.65**	**92.65**	**92.65**
	AUC	0.910	0.927	**0.965**
A4	Accuracy (%)	63.24	**69.12**	61.77
	AUC	0.613	0.686	**0.693**
B2	Accuracy (%)	**58.33**	56.25	47.92
	AUC	**0.625**	0.567	0.524
B3	Accuracy (%)	70.83	**77.08**	70.83
	AUC	0.788	0.792	**0.799**

**Table 7:** Optimal accuracy and $t_{w}$ for $N_{icons}=4$. Temporal parameters for the highlighted set of features are also included.

	Accuracy ($t_{w}^{\mathrm{opt}}$) (%)			$t_{w}^{\mathrm{opt}}$(s)			$t_{\mathrm{on}}$ (s)		$t_{\mathrm{off}}$ (s)	
Subject	Set1	Set2	Set3	Set1	Set2	Set3	${\bar{t}}_{\mathrm{on}}$	$\sigma_{t_{\mathrm{on}}}$	${\bar{t}}_{\mathrm{off}}$	$\sigma_{t_{\mathrm{off}}}$
A1	**92.5**	85.5	83.5	**8**	5	4	2.43	0.8	2.57	1.7
A2	72	69.5	**72**	8	5	**4**	2.74	1.1	1.82	0.5
A3	**93.0**	89.5	78.5	**4**	4	5	3.3	2	1.8	0.3
A4	67.5	**72**	54	15*	**8**	6	3.14	1.1	2.02	0.6
B2	**73**	70	66	**5**	5	4	3.24	1.9	2.5	1.3
B3	**79.0**	62.0	58.0	**5**	5	3	2.6	0.9	2.64	1

## Abbreviations

The following abbreviations are used in this manuscript:

ADD	Attention Deficit Disorder
ADHD	Attention Deficit Hyperactivity Disorder
ALS	Amyotrophic Lateral Sclerosis
AUC	Area under Curve
CP	Cerebral Palsy
BCI	Brain Computer Interface
CFCS	Communication Function Classification System
EMG	ElectroMyoGram
ETI	Eye Tracking Interface
GMFCS	Gross Motor Function Classification System
IQR	Interquartile Range
ITR	Information transfer rate
LDA	Linear Discriminant Analysis
LIS	Locked-in Syndrome
LPFC	Lateral Prefrontal Cortex
NIRS	Near Infrared Spectroscopy
ROC	Receiver Operating Characteristic Curve
RSVP	Rapid Serial Visual Presentation
SMR	Sensorimotor Rhythms
SCP	Slow Cortical Potentials
SSVEP	Steady State Visual Evoked Potential
TBR	Theta Beta Ratio
